# Virus Budding/Host Interactions

**DOI:** 10.1155/2011/963192

**Published:** 2011-10-20

**Authors:** Ronald N. Harty, Anthony P. Schmitt, Fadila Bouamr, Carolina B. Lopez, Claude Krummenacher

**Affiliations:** ^1^Department of Pathobiology, School of Veterinary Medicine, University of Pennsylvania, Philadelphia, PA 19104, USA; ^2^Center for Molecular Immunology and Infectious Disease, Department of Veterinary and Biomedical Sciences, The Pennsylvania State University, University Park, PA 16802, USA; ^3^Laboratory of Molecular Microbiology, NIAID, NIH, Bethesda, MD 20892, USA; ^4^Department of Biochemistry, School of Dental Medicine, University of Pennsylvania, 240 S. 40th Street, Philadelphia, PA 19104, USA

 Viruses have developed unique and oftentimes complex molecular mechanisms to ensure efficient egress of mature virions from infected cells. Over the years, virologists have begun to unravel and appreciate the intricate roles of both viral and host proteins in this process, and particularly the specific recruitment of host factors to promote efficient budding of infectious virus. A better understanding of these virus-host interactions and the mechanisms of virus budding not only will provide fundamental insights into the functions of both viral and host proteins, but also will lead to the emergence of novel strategies to inhibit virion egress and spread. In this special issue on virus budding/host interactions, we have invited both review articles to summarize recent findings in the field as well as original research articles to provide new insights into this late stage of virus replication.

 The first paper of this special issue is a review article that focuses on the current knowledge of arenavirus budding and the critical role that the small RING finger Z protein plays in this process. S. Urata and J. C. de la Torre present a comprehensive discussion on the structure and function of Z and highlight the various L-domain motifs conserved in arenavirus Z proteins and in the matrix proteins of other emerging RNA viruses. Viral L-domains are of particular interest since they mediate recruitment of host proteins to promote virus budding and, as such, constitute attractive and potentially broad-spectrum targets for antiviral drugs designed to inhibit virus egress. Indeed, the authors discuss several strategies and ongoing efforts to identify and screen candidate budding inhibitors. 

 The viral L-domain/host interaction theme continues in the second paper, where Y. Liu et al. have utilized a bimolecular complementation (BiMC) approach to detect, localize, and follow filovirus VP40-host complexes in live mammalian cells in real time. The authors postulate that adaptation of the BiMC approach to the study of virus-host interactions and budding may help address gaps in our understanding of the dynamics, kinetics, and trafficking patterns of virus-host complexes involved in the budding process. In addition, the BiMC approach was used in conjunction with the more established filovirus VP40 virus-like particle (VLP) budding assay to test candidate small molecule inhibitors (identified by *in silico* screening) of L-domain/host interactions for their ability to inhibit particle release. 

 The mechanisms by which viral and host proteins are trafficked and/or targeted to the site of budding are of great interest. The third paper summarizes experimental data in support of a model for assembly and budding of influenza virus from viral bud zones and raft-enriched domains at the plasma membrane. Indeed, assembly and budding at raft domains appears to be a characteristic shared by several RNA virus families. M. Veit and B. Thaa discuss the raft-targeting features of the hemagglutinin (HA) and neuraminidase (NA) glycoproteins of influenza virus, along with the critical roles of M1 and M2 proteins in orchestrating virus assembly and in membrane bending/particle scission, respectively. 

 Little is known about the role of human respiratory syncytial virus (HRSV) glycoproteins during assembly and budding of mature virions. In the fourth paper, M. Batonick and G. W. Wertz address this gap by engineering recombinant viruses and using microscopic and biochemical analyses to demonstrate that deletion of the G and F proteins affected the incorporation of other viral proteins into budding virions; however, their absence did not directly affect the efficiency of virion egress. Thus, this study ascribes a novel role for the G and F glycoproteins during the late stage of HRSV replication.

 In the fifth paper, Snyder et al. describe a novel and intriguing cell lysis system utilized by two archaeal viruses, *Sulfolobus* turreted icosahedral virus (STIV) and *Sulfolobus islandicus* rod-shaped virus 2 (SIRV2). The authors demonstrate that the STIV c92 protein forms unique pyramid-like structures on the cell surface through which newly assembled virions are released during cell lysis. The authors speculate that this new lysis system may be common within other archaeal viral populations present in acidic hot springs. 

*Ronald N. Harty* *Anthony P. Schmitt* *Fadila Bouamr* *Carolina B. Lopez* *Claude Krummenacher*

